# Parent engagement in autism-related care: a qualitative grounded theory study

**DOI:** 10.1080/21642850.2018.1556666

**Published:** 2018-12-15

**Authors:** Stephen J. Gentles, David B. Nicholas, Susan M. Jack, K. Ann McKibbon, Peter Szatmari

**Affiliations:** a Health Information Research Unit, and Offord Centre for Child Studies, McMaster University, Hamilton, Canada; b Faculty of Social Work, University of Calgary, Edmonton, Canada; c School of Nursing, McMaster University, Hamilton, Canada; d Health Information Research Unit, McMaster University, Hamilton, Canada; e Department of Psychiatry and Behavioural Neurosciences, Offord Centre for Child Studies, Hamilton, Canada

**Keywords:** Patient engagement, healthcare consumer, caregiver, parent, autism spectrum disorder

## Abstract

Parents of children with autism assume substantial responsibility for navigating intervention to address autism-related concerns, including involvement in therapy. Little is known, from the perspective of these parents, regarding how to best engage and support them in this navigating process as it evolves over the child’s development. In this article, we present findings from a large qualitative study that investigated how parents of children with autism navigate intervention, to construct an in-depth theoretical account of how this group comes to be engaged in individual-level care. Using grounded theory methods and a symbolic interactionist framework, we analyzed select documents and 45 intensive interviews conducted with 32 mothers and 9 expert professionals from urban and rural regions of Ontario, Canada. Parent-defined *concerns* are the central impetus for the core process of navigating intervention, labeled using parents’ language *making your own way*. We describe how this process is analogous to engaging in care. Four meaning-making processes – *defining concerns*, *informing the self, seeing what is involved*, and *adapting emotionally* – all interacting in an ongoing fashion, together account for parents’ evolving readiness and motivation for taking action to navigate intervention. We illustrate how parents’ readiness and motivation for navigating intervention (and thus for engagement) evolves over a generalized trajectory, according to three highly overlapping processes experienced by most parents: *coming to understand their child has autism*, *going into high gear*, and *easing off*. These findings indicate multiple empirical conditions and factors affecting engagement that service planners and professionals will likely want to consider when seeking parent involvement as a means to improve outcomes in autism. Additionally, theoretical aspects are relevant to the developing understanding of how healthcare consumers in general become engaged in individual care, with implications for patient-centered care.

## Introduction

### The setting of autism

Compared to most types of healthcare consumers, parents of children with autism spectrum disorder (hereafter, autism) are highly motivated and engaged in navigating multiple forms of care in an especially complex context. In this article, we employ findings from a large qualitative study to construct an in-depth theoretical account of how this group becomes engaged in care at the individual level.

Autism is a neurodevelopmental condition whose estimated prevalence, 1 in 68 (Christensen et al., 2016), is not known to be importantly affected by geography, race, or socioeconomic factors (Elsabbagh et al., 2012). The diagnosis is characterized by deficits in social communication and interaction, accompanied by restricted, repetitive patterns of behavior, interests, or activities (American Psychiatric Association, 2013). Despite a core defining set of deficits, the presentation of autism – including behaviors, extent of disability in different areas (intellectual, speech, etc.), and comorbid conditions – is notoriously heterogeneous and child-specific (Anagnostou et al., 2014). Hence the saying, ‘if you’ve met one child with autism, you’ve met one child with autism’ (attributed to advocate Stephen M. Shore). This problem of uniqueness represents a source of complexity, and therefore burden, for parents as they seek to understand the disorder and find solutions to the multiplicity of concerns they attribute to it. Thus, in contrast to many other pediatric health conditions, parents lack a common template for how to respond. This drives them to independently pursue the knowledge they need to inform their action; and parents are known to consult varying information sources in their quest (Gibson, 2017).

As de facto experts on the unique needs of their child, parents are centrally positioned decision makers regarding intervention, broadly defined here as any therapy, service, or modification a parent or care professional considers using to address an autism-related concern. Each of the numerous possible autism-related concerns affecting each child (and family) may be addressed by a multiplicity of available interventions, including behavioral or educational therapies, medications, alternative treatments, financial relief, respite, and environmental modifications. One survey study found parents reported adopting an average of 7 of 111 named treatments for their child (Green et al., 2006). The number and variety of interventions available for consideration adds a second dimension of complexity (besides the disorder itself) that parents must struggle to understand (Grant, Rodger, & Hoffmann, 2015). In Ontario, Canada, families and individuals access wide-ranging publicly funded autism interventions through four provincial government ministries providing diverse services in the domains of health care, child and youth issues, education, and social programs. Additionally, families often pay out of pocket for unfunded private intervention options across similarly wide-ranging domains that include biomedical (including complementary and alternative medicines), educational, clinical, therapist-led, social, and other categories of intervention. Not only do interventions vary widely in terms of their attributes, but also in the amount, quality and credibility of information describing each option (Grant et al., 2015; Offit, 2008). Thus, information represents yet another dimension of complexity.

On the backdrop of this complexity, parents’ search for intervention to address autism-related concerns is often experienced as highly urgent – as it is for most parents of children with serious health concerns. In the case of autism, a highly influential source of urgency is parents’ widespread exposure to the abundant research evidence suggesting that intervening as early as possible is critical to optimizing developmental trajectories for their child, implying better outcomes later in life (Edwards, Brebner, McCormack, & MacDougall, 2016; Vivanti & Dissanayake, 2016). This knowledge represents one of the multiple factors that drives parents, many of whom fear the future implications of autism, to become highly engaged in pursuing intervention. In addition to other external sources of urgency, numerous additional parent-level factors interact uniquely to influence parents’ degree of motivation for pursuing interventions to address the concerns attributable to their child’s autism.

### The focus on patient engagement

Patient engagement has been defined and conceptualized broadly and in different ways (Carman et al., 2013; Center for Advancing Health, 2010; Conway, 2011; McCormack, Thomas, Lewis, & Rudd, 2017). For example, Coulter (2011) defines engagement as centering on the interaction between patients and care professionals, who work to ‘promote and support active patient and public involvement in health and healthcare and to strengthen their influence on healthcare decisions, at both the individual and collective levels’ (p. 10). Others similarly define engagement as occurring at collective levels beyond the individual care level (e.g. the levels of ‘organizational design and governance,’ and ‘policy-making’ (Carman et al., 2013)).

In this paper, we have chosen to focus on engagement more narrowly, at the individual or ‘direct care’ level only (Carman et al., 2013). Since our aim is to represent a patient-centered perspective (where ‘patient,’ includes caregivers or parents), our conceptualization is closer to definitions of those who have positioned engagement as patient-centered rather than an interaction involving health professionals (for example, Center for Advancing Health, 2010; Gruman et al., 2010). Specifically, our study addresses the experience of engagement from the parent’s perspective – a perspective partly situated within clinical settings, but largely outside of them in the ‘lifeworld’ (Barry, Stevenson, Britten, Barber, & Bradley, 2001). Healthcare consumers’ lifeworlds can thus include services and interventions available outside of publicly-regulated systems, and any type of information source they may interact with on their own time including various media (print, broadcast, electronic) and human social connections. In our study, parents became motivated to pursue solutions to health concerns in a context where no one system was equipped to adequately support their needs, so that being engaged involved a necessary degree of independence. Engagement is therefore treated here as a one-sided characteristic, attributable only to the parent (or healthcare consumer), although interactions with care providers and systems as well as other contextual factors are highly relevant, as will be described. Following from these observations of parents’ firsthand experience, we define engagement as an individual healthcare consumer’s readiness and motivation at a given point in time to be involved in navigating intervention to address a health concern. Thus the two processes of navigating and becoming engaged in care are highly related.

Numerous benefits of greater patient engagement have been well established, both at the individual patient level, and the organizational or system levels (Mockford, Staniszewska, Griffiths, & Herron-Marx, 2012; Semrau et al., 2016). For example, at the individual level, being engaged is associated with better care experiences, improved health outcomes, and alignment with many patients’ ultimate preferences for engagement (Blumenthal-Barby, 2017; Greene, Hibbard, Sacks, & Overton, 2013). Key economic benefits have also been established (Charmel & Frampton, 2008; Hibbard & Greene, 2013; Hibbard, Greene, & Overton, 2013). Thus any research that deepens our understanding of the mechanisms of engagement and how it can be fostered is warranted. While interaction-based models of engagement continue to be highly useful in this respect, understanding engagement from a novel firsthand patient-centered perspective may allow for identification of previously unknown individual-level factors and conditions that can be targets for modification. Parents of children with autism represent a particularly informative case because they tend to reach exceptional levels of motivation and engagement due to the pronounced urgency they experience related to their child’s wellbeing. To our knowledge, there has been no other systematic research that provides a holistic empirical explanation of how this group becomes so highly motivated and engaged in care through a process of navigating intervention.

Here we present an in-depth theoretical account of how parents of children with autism come to be engaged in individual care, constructed from the findings of a large qualitative grounded theory study concerned with elaborating parents’ process of navigating intervention from when they first notice signs of the disorder in their child (Gentles, 2015). We begin by introducing the structure of the overall theory, and expand on the elements relevant to a patient-centered understanding of individual-level engagement. We illustrate how parents’ readiness and motivation for navigating intervention (and thus for engagement) evolves over time, first with the early process of coming to understand their child has autism, and later with the processes of going into high gear, and easing off.

## Methods

### Ethics and recruitment

Ethical approval was obtained from the Hamilton Integrated Research Ethics Board prior to recruitment. Recruitment was from diverse sources including two major autism advocacy organizations with broad Ontario reach, a regional board of education, an autism conference, and news publicity. Participants were informed about the study by phone or email, and gave written consent before interviews, after which they received an honorarium. Participant names are replaced by pseudonyms, below.

### Sample size and sampling

The primary data sample comprised 45 in-depth interviews, conducted late 2012–2013; interview participants included 32 mothers (fathers also participating in 3 cases), and 9 professionals with substantial experience supporting parents (4 participants completed a second interview) – representing a comparatively large participant and interview sample for grounded theory (Mason, 2010). Data also included select documents including parent- and professional-authored books – from which instructive passages were typed into notes and coded, and about which memos were written more generally, all with reference to source page ranges.

Participants were intentionally selected from urban and rural regions across Ontario (Leipert, Matsui, Wagner, & Rieder, 2008; Wathen & Harris, 2007), and at different points in their navigating journey to represent maximally varying perspectives. Primarily mothers were chosen to limit sample size needs given available resources (Sandelowski, 1995), and because the literature suggested that their perspective was most useful to represent in an initial study (Gray & Holden, 1992; Mackintosh, Myers, & Goin-Kochel, 2005; Marcenko & Meyers, 1991).

Theoretical sampling was understood according to descriptions of the original grounded theory developers (Charmaz, 2006; Corbin & Strauss, 2008; Glaser, 1978; Glaser & Strauss, 1967; Strauss & Corbin, 1998), as reviewed elsewhere (Gentles, Charles, Ploeg, & McKibbon, 2015). Thus, it was used here as a means to select examples of categories being developed, wherever they may be found, rather than participants per se (Strauss & Corbin, 1998, p. 202), such as by asking new questions in interviews pertinent to underdeveloped categories. Data collection continued until the characteristics of the most important categories were sufficiently developed (Strauss & Corbin, 1998, p. 212).

### Data collection, analysis, and rigor

Grounded theory methods, commonly used for studying new social psychological processes, were used to guide concurrent data collection and analysis – while multiple grounded theory texts were used for guidance (Charmaz, 2006; Corbin & Strauss, 2008; Glaser & Strauss, 1967; Strauss, 1987; Strauss & Corbin, 1998), the primary sub-approach and text followed was Corbin and Strauss (2008), which is arguably consistent with constructivism and symbolic interactionism (Charmaz, 2014; Corbin & Strauss, 2008). Parents completed an initial survey interview (15 minutes) to collect demographic and other attribute data. All participants completed intensive interviews (approximately 90 minutes), conducted either face-to-face or by phone (by SJG), audio-recorded, professionally transcribed, and cleaned. Parents were asked about personal experience (some parents also shared observations of other parents); while professionals were asked for knowledge and observations of parents within their practice (one professional also shared personal experience as parent of a child with autism). Following an iterative flexible interview guide, participants were asked about experiences navigating intervention from the time parents first noticed signs of autism in their child; specific questions evolved with successive interviews according to theoretical sampling.

Data collection and verification of the evolving categories proceeded iteratively according to theoretical sampling informed by ongoing analysis. Analysis consisted of constant comparison employed in coding and category development, analytic memo writing, developing abstract conceptual diagrams, and final integrative writing of the theory, which ensured findings were grounded in primary participant data. All interviews were completely coded, as each provided valuable analytic insights.

The social theory of symbolic interactionism was used as an explicit framework guiding analysis; briefly, it assumes that people respond emotionally or through action to the meanings they hold for the things in their situation, which they construct through social interactions and modify through an interpretive process (Blumer, 1969). Grounded theory methods and a symbolic interactionist framework are well suited to identifying relevant informative factors and conditions underlying individual-level engagement from a patient-centered standpoint because they both prioritize firsthand person-centered perspectives.

Member checking was achieved in a manner consistent with grounded theory by gauging participants’ reactions regarding categories’ coherence with parent experience; and where there was coherence, directing subsequent discussion to generate new properties of those categories (Charmaz, 2006, p. 111). A detailed account of reflexivity methods used, and of the researcher position with respect to the research is available elsewhere (Gentles, Jack, Nicholas, McKibbon, & Szatmari, 2014). Data management and analysis were supported by software (NVivo 10; QSR).

## Results

### Participant characteristics

**Figure 1.**
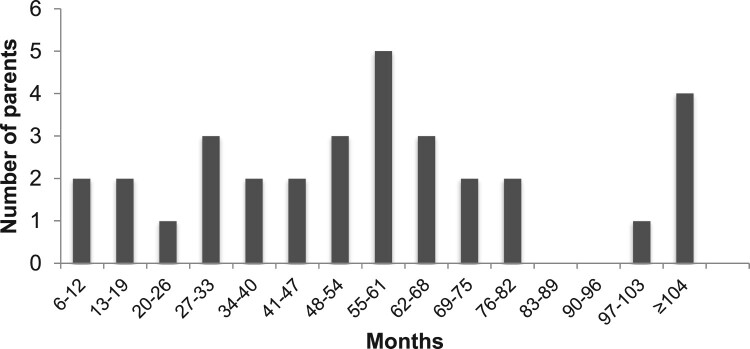
Time spent navigating intervention at point of interview, since first concern of autism.

The 32 parents interviewed varied widely in several characteristics relevant to the experience of navigating intervention. Severity of children’s autism ranged from high-functioning to severe; child ages ranged from 2.5–13.0 years (one child 18 years); and 13% of families had multiple children with autism. Ages at diagnosis ranged from 20 months to 10 years, but had a skewed distribution (mode 25 months, median 36 months). Age at first concern ranged from 7–72 months (median 23 months); lag times between first concern and diagnosis were mostly 2–23 months (median 12, maximum 72 months). An important characteristic is the distribution of times parents had been navigating intervention at the point they were interviewed (Figure 1), which ranged from 1 year (2 parents) to over 8 years (5 parents). This variation was key to analyzing evolution of the process presented.

Geographically, parents were from urban (78%) and rural (22%) areas, covering 13 of Ontario’s 51 regions. Of 32 mothers, 20 were daytime caregivers, 13 were fulltime and 6 part-time employed. Education ranged from high school diploma (3) to university graduate degree (5). Ethnic origin (11), aboriginal status (1), or religion (1) were additional factors that emerged as relevant during interviews.

The 9 professionals interviewed had frontline experience in roles of autism agency manager, family support organization manager, special education teacher, educational director, social worker, behavioral therapist, occupational therapist, speech and language pathologist, diagnostician, nurse practitioner, clinical psychologist, and psychiatrist; and worked in varying urban and rural regions of Ontario.

### Overview of the theoretical process of ‘making your own way’

**Figure 2.**
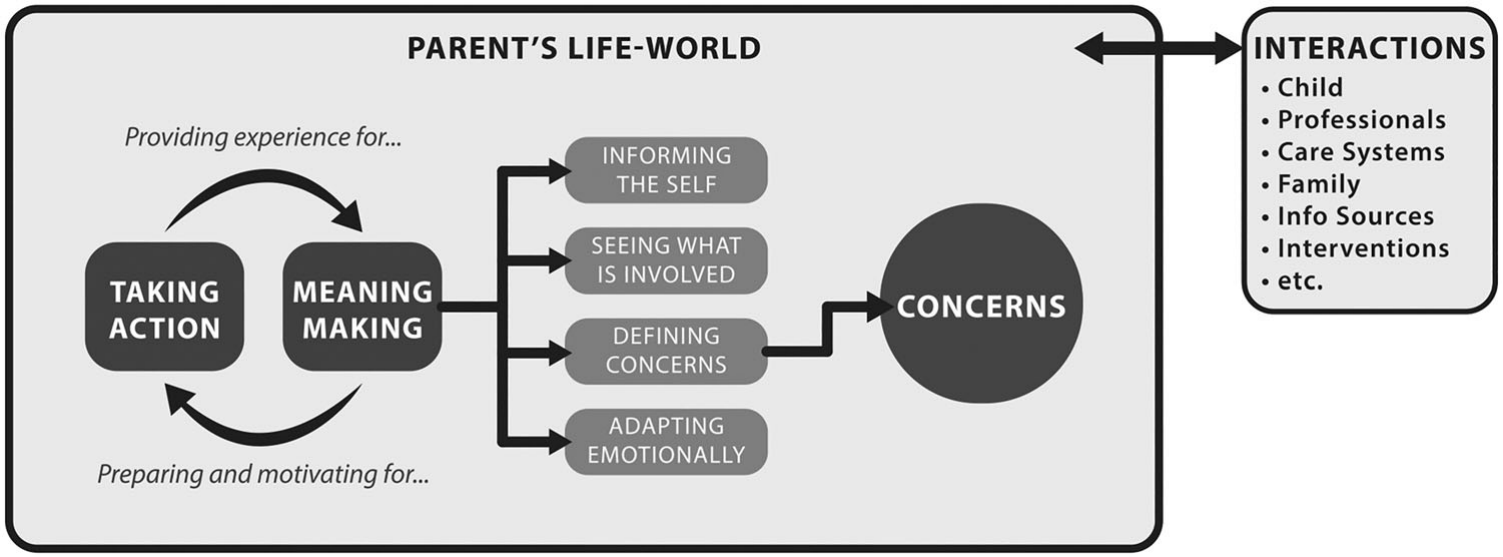
The process of ‘Making your own way’ the within context of a parent’s lifeworld.

Elements of the general theoretical process of navigating intervention that are specifically relevant to understanding the mechanisms underlying parents’ engagement in care are represented in Figure 2. The parent’s lifeworld is an experiential space filled with social and psychological interactions, both with other people and things (the child with autism, professionals, care systems, other family members, information sources, interventions, etc.), and with the self. The process of navigating intervention, labeled in parent participants’ language *making your own way*, encompasses two mutually-influencing processes: meaning-making and taking action. *Concerns* are the most influential product of parents’ meaning-making, providing the source impetus for parents’ action directed at navigating intervention; thus, *concerns* are positioned at the center of the parent’s lifeworld.

The overall meaning-making process, through which parents adjust to their situation of having to navigate intervention, consists of four sub-processes: *defining concerns* related to autism, *informing the self*, *seeing what is involved* in taking action, and *adapting emotionally*. Elaborated below, these sub-processes prepare and motivate parents for taking action to navigate intervention. Relevant action includes both *pursuing intervention* and *pursuing information*; both generate new experience and knowledge, which informs further meaning-making.

### Concerns: the impetus for navigating and engagement

Concerns were defined for purposes of this study as any circumstance or condition attributable to a child’s autism that the parent perceives as sufficiently problematic to want to take personal action to address. They may affect the child, parent, or family. Thus, by definition, concerns motivate parents’ action within the process of navigating intervention. Notably, while action is often motivated by the problematic aspects of concerns, it can also be motivated by positive aspects including concerns for further happiness, or hope for even better outcomes after witnessing initial progress in one’s child.

Parents generally described numerous concerns for any one child. The number and nature of concerns invariably evolved for parents whose children aged through developmental transitions. Importantly, parents described multifaceted diversity in the autism-related problems they personally defined as concerns.

Different perspectives or frameworks can be adopted to categorize and interpret this diversity. For example, the World Health Organization’s (2001) International Classification of Functioning, Disability and Health (ICF), while not used in our analysis, can account for most parent-described concerns directly affecting the child with autism by classifying functioning into three levels: body or body part, whole person (activity), or whole person in a social context (participation) (World Health Organization, 2002). Parents described concerns in each category, with greatest priority and urgency generally placed on concerns affecting activity and participation.

These child-specific concerns ranged from highly specific, and often related to body-level functioning (including sensory, motor, sleep, behavioral, affective mental health, digestive and nutritional), to more general and often relating to activity- or participation-level functioning (including social, communication, toileting, self care, academic, personal relationships, community integration, vocational, independent living, autonomy, and self-advocacy). Comorbid disorders, common in autism, were another type of concern (e.g. epilepsy, cerebral palsy, infection, anxiety). While the Ontario parents interviewed described the overall disorder, autism, as a concern because of the more specific (often functional) concerns it gave rise to, almost none described its fundamental presence as a modifiable concern warranting their action to eliminate. This was partly because most parents understood that there was no way to ‘fix’ or ‘cure’ autism (by contrast to specific functional concerns, which were generally perceived as treatable), or because they had come to view autism itself as not intrinsically undesirable given its importance to their child’s identity including strengths.

The most general overarching concern that parents commonly articulated was for their child’s happiness, both in the present and long-term future. The key challenges to this related to both social connectedness (for example, the desire that an adult child could have interested friends who could look out for them) and autonomy (self-awareness of, and the ability to communicate and defend their own interests). Unlike worries that parents of typically developing children are likely to experience regarding their child’s happiness, parents in this study described profoundly difficult situations that made their child’s happiness a highly uncertain outcome. One mother’s anecdote illustrates the acute anguish that can accompany a parent’s fundamental concern for their child’s connectedness in the world:

It was my husband, came downstairs one night and he was crying. And he said our son had said his prayers that night and prayed for friends. And, he said that hurt more than hearing him say, ‘I wish I was dead.’ Because there’s nothing worse in the world than wanting your kid … [knowing that] your kid wants to have friends.

#### Secondary concerns

While the ICF may effectively capture child-specific concerns – directly affecting the *individual* with the diagnosis – it does not account for autism-related concerns affecting the parent or family. This is significant since, from many parents’ perspective, it is not just the child who has autism. Parents reflected this in explicit statements – for example, ‘The day that a child gets diagnosed with autism, the whole family is diagnosed with it, in my opinion.’ It was also apparent from their implicit language, such as using ‘we’ to refer to events affecting the child, like being diagnosed. As one highly experienced professional commented, ‘We tend to think of the kid, and what the kid’s strengths and difficulties are. But really we need to see an entire system, and the family’s interaction, and the quality of their life, etcetera.’ The parent’s perspective, as embodied by findings in this study, implies that concerns should encompass anything affecting the interdependent *autism unit* – i.e. extending to the supportive family affected by the child’s autism (e.g. parents, siblings).

Secondary autism-related concerns (affecting parents or family) usually evolved from primary concerns (affecting the child) in several ways. First, parents could be directly affected by primary concerns. Two characteristic examples illustrate how primary concerns can produce physical and emotional concerns in the parent. In one example (physical concerns), Salome had a 5-year-old son with severe autism whose earlier sleeplessness and need for constant supervision had led to her chronic sleep deprivation – ‘I’m watching him by seconds, not by minutes … I don’t know if you will believe me, but by *milliseconds*, just trying to watch him, or playing with him, or teaching him anything.’ Salome reported surviving on one or two hours of sleep a night for several years, during which she was concerned for her family’s ability to work and go to school, and her own ability to function. The second example (emotional concerns) is the intense grieving that some parents described passing through after discovering their child had autism. At least one parent described seeking the help of a clinical psychologist in an attempt to cope with resulting personal mental health concerns.

Additionally, parents were significantly affected by the considerable *burdens* associated with navigating interventions – which are important to distinguish from burdens of parenting or coping with child behaviors. Briefly, navigating-related burdens include both stress, and the loss of expendable resources attributable to the work of navigating intervention (described further in Gentles, 2015). Stress precipitated secondary concerns such as depression, anxiety, marital breakdown, and acute and chronic need for respite or relief. The combined work navigating all interventions to address the multiplicity of autism-related concerns was considerable for most parents. The loss of expendable resources associated with this work – including physical and emotional energy, time, and financial resources – could be a source of serious downstream personal concerns. These included personal bankruptcy or financial insecurity, chronic sleep deprivation and other forms of physical and emotional exhaustion impairing capacity to function, deterioration of physical health due to lack of self-care, family dysfunction and breakdown, exacerbated stress and affective disorders, and additional emotional problems due to guilt for neglecting family or not doing enough for the child.

It is important to be attentive to the mechanisms by which secondary concerns come about, since they create the need for interventions to address family wellbeing – and additional navigating work to obtain them. We now briefly describe the four meaning-making sub-processes (Figure 2) to illustrate how they prepare and motivate parents to take the action related to navigating intervention to address the concerns described above – action manifesting as engagement in care.

### Sub-processes underlying readiness and motivation for engagement

These four sub-processes are highly interrelated, occurring across parents’ trajectories navigating intervention.

*Defining concerns* is a process by which the parent (or healthcare consumer) comes to perceive and ascribe deep personal meanings to an autism- or health-related problem that is facing them; and these meanings necessarily motivate them to take action to address or resolve it. Many parents in this study began defining *specific* concerns motivating their help-seeking prior to understanding their child had autism, the most common first concern being delayed speech. Eventually, parents defined the more general, overarching developmental concern of autism, often as a result of defining related specific concerns (see *Coming to understand the child has autism*, below). Importantly, the meanings underlying such concerns are constructed through the inextricably linked processes of *informing the self* and *adapting emotionally*.

*Informing the self* involves interacting with different information sources to prepare oneself with the knowledge and understanding about the concern or about options for addressing it (intervention) to better direct one’s actions. It includes passive and active interactions with text-based sources (books, internet, etc.), people (professionals, relatives, other parents, acquaintances), observations (e.g. of child) and experiential learning (e.g. understanding demands of the system). *Adapting emotionally* was integral to the meanings parents attached to the knowledge gained by *informing the self*.

*Adapting emotionally* to implications parents see for themselves and their child after reflecting on their situation includes several specific sub-processes (Table 1). Difficulty with these aspects generally delayed parents’ readiness and motivation for navigating intervention (i.e. engagement). Thus, struggling with accepting the possibility of autism through the emotionally self-protective mechanism of denial delayed parents in *knowing something is wrong* (see *Coming to understand the child has autism*) and even produced obstructiveness to action*.* Meanwhile, difficulty *releasing culturally-based hopes and expectations for their child’s future* after imagining the possible long-term implications (e.g. for marriage, employment, living independently) or *accepting an uncertain and fearful future for their child* related to the vague images and expectations often acquired, could lead parents to feel overwhelmed, sometimes paralyzed with grief, and incapable of action for varying durations. Later, difficulty *surviving the emotional strains of making your own way* – in which parents resorted to remarkable social and psychological strategies to endure and cope with the often heartrending challenges (causing sadness, despair, fear, or anxiety) that arise from the stress-related obstacles and burdens encountered when navigating intervention – could lead to becoming overwhelmed and loss of emotional functioning needed for navigating intervention efficiently. Lastly, trouble with *redefining one’s role and self according to new occupational requirements –* where parents demonstrated striking adaptability (expanding skills, going outside comfort zones, acting against their nature, giving up actual and hoped for social identities, and reaching beyond their skillset) to meet the requirements of their involuntary new roles navigating intervention – could impair parents’ ability and sense of competence for meeting system demands and advocating effectively. Successfully adapting emotionally in these ways, meanwhile, helped prepare and motivate parents for proactive engagement.

**Table 1.** Aspects of the process of *adapting emotionally*.

	Examples of difficulties
Earlier aspects	
*Accepting the possibility of autism*	I [father] would convince myself that … I’d read something about autism that, you know, in retrospect, seemed to square exactly with her behavior. And then I would just sort of try and find those exceptions. And I’d think, ‘Oh! But, you know, she laughed that one time. Kids with autism don’t have a sense of humour.’ Or, you know, ‘She hugged me. So, kids with autism aren’t affectionate. So it couldn’t be autism.’
*Releasing culturally-based hopes and expectations for the child’s future*	But for [my husband and me], when we’ve talked about it since, we grieved for the kids we thought we were getting. You know, you think you’re getting your neurotypical, normal children that are going to run and play. You have this idea in your head of how they’re going to grow up, and the things that you’re going to do with them. And when somebody tells you, ‘Oh, they might have autism … ’ all those things are sort of ripped away from you. And you have to grieve those pictures in your head that you’re never going to be able to do with them. Or, that’s what we thought then. I mean, that’s not really the reality of it. So we went through this starting of a grieving process.
*Accepting an uncertain and fearful future for their child*	The hardest part has always been fear for his future … It’s an ongoing thing … what’s going to happen to him when I can’t be there anymore? Fearing that nobody can give him what he needs if I’m not there to advocate for him and be his voice, because he’s non-verbal. So that’s a huge fear. Fear of him possibly being abused, or put into a situation where he can’t speak for himself, and something happening. And how would anybody ever know?
Later aspects	
*Surviving the emotional strains of navigating*	If I do the best I can, at the end of the day I just have to be at peace with that. Because if I don’t take care of myself, they’ve got nothing, because I’m all they’ve got. So I have to be at peace with what I’m able to do. And if I have a bad day where I don’t do much – oh well, so does everybody! … I give myself permission to not be perfect, I guess.
*Redefining one’s roles and self according to new occupational requirements*	You know, they say you can advocate for your child – you’re your child’s best advocate. Well to me, advocate just is another word for bitch. Because as soon as you start advocating for your child, well then you’re seen as that parent: ‘Oh, here comes that parent again.’

*Seeing what is involved* in taking action to address concerns occurs when parents experience the work involved in navigating intervention and learn about the systems of care they must interact with. Parents thus gain concrete ideas of what is required to overcome obstacles to intervention. They also usually learn that ‘it is up to me’ to advocate for their child. Together, this prepares and motivates them to be highly proactive about engaging in care.

### Long-term trajectory of engagement

**Figure 3.**
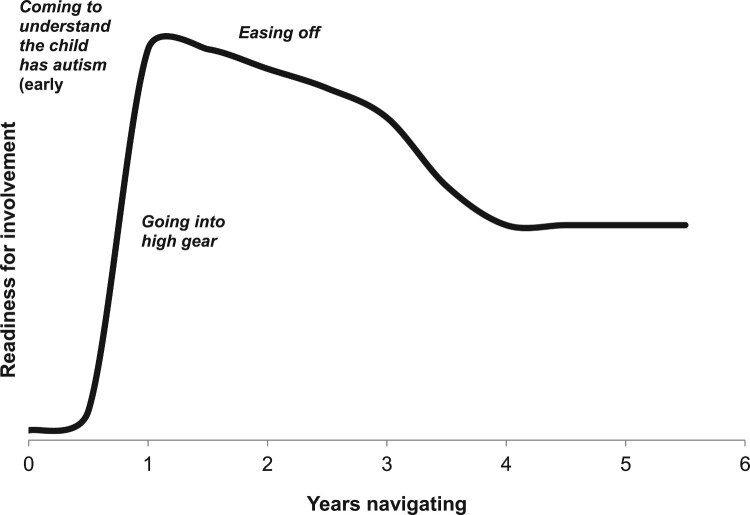
Hypothetical typical long-term trajectory of readiness for involvement.

Parents’ capacity and motivation for autism-related action evolve in importantly different ways and at different rates over a parent’s lifetime according to three processes (a hypothetical case consistent with participants’ more common accounts is represented in Figure 3):

• Coming to understand the child has autism

• Going into high gear

• Easing off

While these processes generally each begin in the sequence listed, they are highly overlapping and interrelated, often with no identifiable endpoint. Nevertheless they provide a useful means to understand the long-term trajectory of parents’ level of engagement over the lifelong course of autism.

*Coming to understand the child has autism* is the first process within the greater evolving process of navigating intervention. It is also the most significant example of the sub-process of *defining concerns*, where the parent initially defines, and becomes motivated to address, the overarching developmental concern of autism itself. Notably, most parent participants reached awareness before the official diagnosis. Four flexible steps (some parents skipping earlier steps) account for the highly variable paths parents take to initial awareness. First, in *forming an image of difference*, parents respond to initial perceptions of apparently minor signs by seeing their child as slightly different, but in a non-worrisome way, while observing how signs evolve with development. Second, in *starting to question the signs*, parents respond to emerging vague suspicions that a sign may be problematic by taking some action by seeking further information and recording observations to identify a potential problem. Third, parents progress to *knowing something is wrong* when they interpret what they have learned to indicate a serious problem, and become urgently motivated to quickly understand the problem enough to know *how* to intervene and begin accessing intervention that might be indicated. The following quote illustrates the time that can sometimes be required in transitioning to *knowing something is wrong*.

Actually, I worried for a long time because I was telling myself, ‘No, it’s going to happen next month. He’ll talk next month. It’s gonna be next month,’ you know. I knew something was wrong. But then I was telling myself, ‘You know what, maybe it’s a little too early. You know, kids, sometimes they develop in different ways. So maybe he’s taking a little longer. He will talk. He will talk.’ That’s what I was telling myself. But then I said, ‘Uh-oh, that’s it. We have to do something now.’

Finally, *being convinced it is autism* occurs either after the parent integrates the necessary information themselves, or (more rarely) after being informed decisively by a professional, both of which usually increase parents’ urgency and motivation to pursue intervention, sometimes after a period of being emotionally overwhelmed. Importantly, this is a process when parents’ motivation and capacity for action generally increases at a gradual rate due to the many potential delays to reaching full understanding and acceptance – described in more detail elsewhere (manuscript submitted).

*Going into high gear* involves parents responding to their sense of urgency about their child’s autism by entering a mode, lasting months to several years, of powerful motivation for taking action to pursue intervention in which they expend personal resources (time, energy, and money) at a rate that is unsustainable in the long term. As described by one parent,

I automatically went into the ‘fix’ mode, you know, got to do something now … He needs therapy right now. I can’t just sit there and let him go down this path … I clicked … The next part was finding out what I had to do, and finding that information.

While this process started at various points in parents’ journeys, it first occurred for most parents early, soon after *coming to understand the child has autism*. Specific early triggers included learning the importance of intervening early, seeing one’s child struggle, and other sources of anxiety about possible future outcomes of autism. At later points, urgency-related triggers included encountering obstacles to funded intensive behavioral intervention parents felt necessary for their child’s developmental progress, or crises involving child well-being or unmanageable behavior. While parents had surprisingly high levels of capacity for action, the resource loss caused by continuing in this mode eventually resulted in significant stress, fatigue, and exhaustion for many parents, which reduced this capacity and forced them to scale back.

*Easing off* is a process where parents respond to implications of knowing their child will always have autism, and navigating intervention will be a lifelong process as new concerns continue to arise with development into adulthood. The process generally involves parents modifying their approach to navigating intervention by restoring balance in their lives. Restoring balance, in turn, entails reducing the intensity of pursuing intervention by expending personal resources at a more sustainable rate – an analogy shared by several parents was, shifting to marathon instead of sprint pace. While easing off generally requires a perspective gained from prolonged or repeated experience navigating intervention, it includes any response to concerns resulting from neglected aspects or one’s life or family due to a child’s special needs. Thus, many parents began this process in some way even early in their journey, after reflecting on their situation. The following mother described this as a shared intentional process involving her husband:

It was like, OK, we’ve been doing this for a really long time. We’ve done the very best that we can for our kids and we’re tired. And it would be nice to take some time off – like a real weekend, like other people have where we’re not running around like maniacs getting up at 5:00 in the morning … And so we had made all of the conscious decisions that we were ready to start slowing down with the services that were provided to us, and that we were ready to take a break.

The effect of *easing off* on engagement generally manifests as decreasingly intense motivation for action. Simultaneously, it results in a healthier parent with a more sustainable capacity for involvement in intervention in the long run, and continued ability to respond to urgent child needs stemming from new concerns that arise over time.

## Discussion

In contrast to earlier work that has addressed engagement in individual care by parents of children with autism as a static phenomenon (Valentine, 2010), we here characterize engagement as a process that evolves according to parents’ changing motivation and readiness for navigating intervention starting from when they first perceive signs of delay in their child. In this respect, our findings partially fit with those of another qualitative study in autism (Fleischmann, 2005), which defined a ‘period of readjustment’ (analogous to our *meaning-making* process) that similarly ‘prepared [parents] for action’ (p. 299), although this was only described *after* diagnosis.

Other authors have used the idea of stages to understand the experiences of parents of children with autism. DePape and Lindsay (2015) applied the Family Life-Cycle Model (Carter & McGoldrick, 1988) in their meta-synthesis of 31 qualitative studies to derive 6 themes representing stages (pre-diagnosis, diagnosis) and experiences parents passed through. While organizing findings in terms of stages has merit because it allows some understanding of process, rigidly defined stage theories have been heavily criticized for their failure to account for empirical variation (e.g. Stroebe, Schut, & Boerner, 2017; West, 2005). We therefore emphasize that ours is not a stage theory; and we intentionally used the term ‘stage’ loosely to refer only to what is invariably the initial process: *coming to understand the child has autism*. Further, we recommend that stages, themes or other high-level qualitative categories of parent experience should generally not be referenced to external events such as diagnosis, given our findings that parents’ meaning-making processes only partially map to such events.

The Ontario services context has changed since data collection for this study. In June 2017, the provincial ministry of children and youth services launched the new Ontario Autism Program supported by a major funding increase. This program is intended to fill numerous gaps, including reducing wait-times, expanding services to more children across development than were covered by the previous program, and starting intervention at younger ages (Ontario Ministry of Children and Youth Services, 2017). Thus the experiences of new parents even prior to diagnosis may differ from those interviewed for this study. Evidence-based interventions are being piloted in some Ontario service areas including parent-mediated intervention like the Social ABCs (Brian, Smith, Zwaigenbaum, & Bryson, 2017). Our findings suggest that the high levels parent-reported satisfaction and acceptability of this intervention (Brian, Smith, Zwaigenbaum, Roberts, & Bryson, 2015) may be partly attributable to the fact that it meets parents’ powerful need and motivation for involvement at an appropriate time.

Lastly, this study provides an important empirical example of healthcare consumer perspectives that we believe has potential implications for shared decision making. We have not only elaborated how decisions center on the person (rather than the clinical encounter), but also established how decisions derive specifically from the *concerns* people construct. This observation may be transferable to other patient and caregiver groups who are similarly engaged enough to define their own concerns. We thus propose there is utility in increasing focus within clinical settings on such patient-defined *concerns* – i.e. upstream of *decision points*, which are the typical focus in shared decision making – as a fundamental strategy to meet the identified need for greater patient-centeredness in decisional support provided to healthcare consumers (Clayman, Gulbrandsen, & Morris, 2016).

### Limitations and directions for future work

While many aspects of the findings presented here may be highly transferable to different groups of parents of children with autism across Western cultures, caution is warranted. Specifically, in focusing this initial study of care navigation on the experience and responses of mothers of predominantly younger children with autism, the findings do not sufficiently account for potentially important variations in the experience and responses of several other key groups that face unique challenges related to navigating care in autism: families in northern and remote regions, parents for whom English is a second language, fathers, and parents of adult children. Sufficiently addressing the variations among these groups was beyond the scope and resources for this study. There is growing recognition, meanwhile, how each group merits greater attention. For example, a recent major Canadian needs assessment survey recommended targeted outreach to address unique issues faced in northern communities, and among those with linguistic challenges (Lai & Weiss, 2017; Weiss, Whelan, McMorris, & Carroll, 2015). Similarly, despite early calls for research on fathers of children with autism (Rodrigue, Morgan, & Geffken, 1992), Braunstein, Peniston, Perelman, and Cassano (2013) found this group continues to be neglected; meanwhile, a recent meta-synthesis highlighted the dearth of credible qualitative research on fathers (Lashewicz, Shipton, & Lien, 2017). We therefore propose that separate studies, with dedicated recruitment efforts, are highly warranted to inform strategies for catering to each population’s unique needs.

Explicit conceptualization of engagement as an individual level phenomenon was useful for revealing important factors from an in-depth first-person perspective. This approach, however, fails to systematically account for factors influencing engagement at higher levels. Recent literature has promoted the adoption of comprehensive integrated multi-level strategies to promote patient engagement (McCormack et al., 2017). Thus it is advisable, when developing interventions for improving parent engagement, to also consider knowledge of broader organizational and societal barriers when possible (Carman et al., 2013).

## References

[CIT0001] American Psychiatric Association. (2013). *Diagnostic and statistical manual of mental disorders, Fifth Edition (DSM-5)*. Washington, DC: Author.

[CIT0002] Anagnostou, E., Zwaigenbaum, L., Szatmari, P., Fombonne, E., Fernandez, B. A., Woodbury-Smith, M., … Scherer, S. W. (2014). Autism spectrum disorder: Advances in evidence-based practice. *Canadian Medical Association Journal*, *186*(7), 509–519. doi: 10.1503/cmaj.121756 24418986 PMC3986314

[CIT0003] Barry, C. A., Stevenson, F. A., Britten, N., Barber, N., & Bradley, C. P. (2001). Giving voice to the lifeworld. More humane, more effective medical care? A qualitative study of doctor-patient communication in general practice. *Social Science & Medicine*, *53*(4), 487–505. 11459399 10.1016/s0277-9536(00)00351-8

[CIT0004] Blumenthal-Barby, J. S. (2017). ‘That’s the doctor’s job’: Overcoming patient reluctance to be involved in medical decision making. *Patient Education and Counseling*, *100*(1), 14–17. doi: 10.1016/j.pec.2016.07.010

[CIT0005] Blumer, H. (1969). *Symbolic interactionism: Perspective and method*. Los Angeles, CA: University of California Press.

[CIT0006] Braunstein, V. L., Peniston, N., Perelman, A., & Cassano, M. C. (2013). The inclusion of fathers in investigations of autistic spectrum disorders. *Research in Autism Spectrum Disorders*, *7*(7), 858–865. doi: 10.1016/j.rasd.2013.03.005

[CIT0007] Brian, J. A., Smith, I. M., Zwaigenbaum, L., & Bryson, S. E. (2017). Cross-site randomized control trial of the social ABCs caregiver-mediated intervention for toddlers with autism spectrum disorder. *Autism Research**,* 10, 1700–1711. 28574669 10.1002/aur.1818

[CIT0008] Brian, J. A., Smith, I. M., Zwaigenbaum, L., Roberts, W., & Bryson, S. E. (2015). The social ABCs caregiver-mediated intervention for toddlers with autism spectrum disorder: Feasibility, acceptability, and evidence of promise from a multisite study. *Autism Research*, *9*(8), 899–912. doi: 10.1002/aur.1582 26688077 PMC5064621

[CIT0009] Carman, K. L., Dardess, P., Maurer, M., Sofaer, S., Adams, K., Bechtel, C., & Sweeney, J. (2013). Patient and family engagement: A framework for understanding the elements and developing interventions and policies. *Health Affairs*, *32*(2), 223–231. doi: 10.1377/hlthaff.2012.1133 23381514

[CIT0010] Carter, B., & McGoldrick, M. (1988). *The changing family life cycle: A framework for family therapy* (2nd ed.). New York, NY: Gardner Press.

[CIT0011] Center for Advancing Health. (2010, April 09). *A new definition of patient engagement: What is engagement and why is it important.* Engagement Behavior Framework, 1–17. Retrieved from http://www.cfah.org/pdfs/CFAH_Engagement_Behavior_Framework_current.pdf

[CIT0012] Charmaz, K. C. (2006). *Constructing grounded theory: A practical guide through qualitative analysis*. London: Sage.

[CIT0013] Charmaz, K. C. (2014). *Constructing grounded theory* (2nd ed.). Thousand Oaks, CA: Sage.

[CIT0014] Charmel, P. A., & Frampton, S. B. (2008). Building the business case for patient-centered care. *Healthcare Financial Management: Journal of the Healthcare Financial Management Association*, *62*(3), 80–85. 19097611

[CIT0015] Christensen, D. L., Baio, J., Van Naarden Braun, K., Bilder, D., Charles, J., Constantino, J. N., ... CDC, C. f. D. C. a. P. (2016). Prevalence and characteristics of autism spectrum disorder among children aged 8 years–autism and developmental disabilities monitoring network, 11 Sites, United States, 2012. *MMWR Surveillance Summaries*: *Morbidity and mortality weekly report. Surveillance summaries/CDC*, *65*(3), 1–23. doi: 10.15585/mmwr.ss6503a1 PMC7909709 27031587

[CIT0016] Clayman, M. L., Gulbrandsen, P., & Morris, M. A. (2016). A patient in the clinic; a person in the world. Why shared decision making needs to center on the person rather than the medical encounter. *Patient Education and Counseling*, *100*(3), 600–604. 27780646 10.1016/j.pec.2016.10.016

[CIT0017] Conway, J. B. (2011). Public and patient strategies to improve health system performance. In L. Olsen, R. S. Saunders, & J. M. McGinnis (Eds.), *Patients charting the course: Citizen engagement and the learning health system* (pp. 103–117). Washington, DC: National Academies Press.

[CIT0018] Corbin, J., & Strauss, A. (2008). *Basics of qualitative research: Techniques and procedures for developing grounded theory* (3rd ed.). Thousand Oaks, CA: Sage.

[CIT0019] Coulter, A. (2011). *Engaging patients in healthcare*. New York, NY: McGraw-Hill Education.

[CIT0020] DePape, A. M., & Lindsay, S. (2015). Parents’ experiences of caring for a child with autism spectrum disorder. *Qualitative Health Research*, *25*(4), 569–583. doi: 10.1177/1049732314552455 25246329

[CIT0021] Edwards, A., Brebner, C., McCormack, P., & MacDougall, C. (2016). The early intervention message: Perspectives of parents of children with autism spectrum disorder. *Child: Care, Health &amp; Development*, *43*(2), 202–210. doi: 10.1111/cch.12428 27891656

[CIT0022] Elsabbagh, M., Divan, G., Koh, Y.-J., Kim, Y. S., Kauchali, S., Marcín, C., … Fombonne, E. (2012). Global prevalence of autism and other pervasive developmental disorders. *Autism Research*, *5*(3), 160–179. doi: 10.1002/aur.239 22495912 PMC3763210

[CIT0023] Fleischmann, A. (2005). The hero’s story and autism: Grounded theory study of websites for parents of children with autism. *Autism*, *9*(3), 299–316. doi: 10.1177/1362361305054410 15937044

[CIT0024] Gentles, S. J. (2015). *Making your own way: A grounded theory study of how parents of children with autism navigate intervention* (Doctoral thesis). McMaster University, Hamilton, Ontario, Canada. Retrieved from http://hdl.handle.net/11375/18077

[CIT0025] Gentles, S. J., Charles, C., Ploeg, J., & McKibbon, K. A. (2015). Sampling in qualitative research: Insights from an overview of the methods literature. *The Qualitative Report*, *20*(11), 1772–1789.

[CIT0026] Gentles, S. J., Jack, S. M., Nicholas, D. B., McKibbon, K. A., & Szatmari, P. (2014). A critical approach to reflexivity in grounded theory. *The Qualitative Report*, *19*(44), 1–14.

[CIT0027] Gibson, A. N. (2017). A survey of information source Preferences of parents of individuals with autism spectrum disorder. *Journal of Autism and Developmental Disorders*, *47*(7), 2189–2204. doi: 10.1007/s10803-017-3127-z 28451948

[CIT0028] Glaser, B. G. (1978). *Advances in the methodology of grounded theory: Theoretical sensitivity*. Mill Valley, CA: The Sociology Press.

[CIT0029] Glaser, B., & Strauss, A. (1967). *The discovery of grounded theory*. Chicago, IL: Aldine.

[CIT0030] Grant, N., Rodger, S., & Hoffmann, T. (2015). Intervention decision-making processes and information preferences of parents of children with autism spectrum disorders. *Child: Care, Health & Development*, *42*(1), 125–134. doi: 10.1111/cch.12296 26489390

[CIT0031] Gray, D. E., & Holden, W. J. (1992). Psychosocial well-being among the parents of children with autism. *Australia and New Zealand Journal of Developmental Disabilities*, *18*(2), 83–93.

[CIT0032] Green, V. A., Pituch, K. A., Itchon, J., Choi, A., O’Reilly, M., & Sigafoos, J. (2006). Internet survey of treatments used by parents of children with autism. *Research in Developmental Disabilities*, *27*(1), 70–84. doi: 10.1016/j.ridd.2004.12.002 15919178

[CIT0033] Greene, J., Hibbard, J. H., Sacks, R., & Overton, V. (2013). When seeing the same physician, highly activated patients have better care experiences than less activated patients. *Health Affairs*, *32*(7), 1299–1305. doi: 10.1377/hlthaff.2012.1409 23836747

[CIT0034] Gruman, J., Rovner, M. H., French, M. E., Jeffress, D., Sofaer, S., Shaller, D., & Prager, D. J. (2010). From patient education to patient engagement: Implications for the field of patient education. *Patient Education and Counseling*, *78*(3), 350–356. doi: 10.1016/j.pec.2010.02.002 20202780

[CIT0035] Hibbard, J. H., & Greene, J. (2013). What the evidence shows about patient activation: Better health outcomes and care experiences; fewer data on costs. *Health Affairs*, *32*(2), 207–214. doi: 10.1377/hlthaff.2012.1061 23381511

[CIT0036] Hibbard, J. H., Greene, J., & Overton, V. (2013). Patients with lower activation associated with higher costs: Delivery systems should know their patients’ ‘scores’. *Health Affairs*, *32*(2), 216–222. doi: 10.1377/hlthaff.2012.1064 23381513

[CIT0037] Lai, J. K. Y., & Weiss, J. A. (2017). Priority service needs and receipt across the lifespan for individuals with autism spectrum disorder. *Autism Research*, *19*, 774–712. 10.1002/aur.1786 PMC5573942 28383156

[CIT0038] Lashewicz, B. M., Shipton, L., & Lien, K. (2017). Meta-synthesis of fathers’ experiences raising children on the autism spectrum. *Journal of Intellectual Disabilities*, *69*(2), 1–15. 10.1177/1744629517719347 28705095

[CIT0039] Leipert, B. D., Matsui, D., Wagner, J., & Rieder, M. J. (2008). Rural women and pharmacologic therapy: Needs and issues in rural Canada. *The Canadian Journal of Rural Medicine*, *13*(4), 171–179. 18845069

[CIT0040] Mackintosh, V., Myers, B., & Goin-Kochel, R. (2005). Sources of information and support used by parents of children with autism spectrum disorders. *Journal on Developmental Disabilities*, *12*, 41–51.

[CIT0041] Marcenko, M., & Meyers, J. C. (1991). Mothers of children with developmental disabilities: Who shares the burden? *Family Relations*, *40*(2), 186–190.

[CIT0042] Mason, M. (2010). Sample size and saturation in PhD studies using qualitative interviews. *Forum: Qualitative Social Research*, *11*(3), 8.

[CIT0043] McCormack, L., Thomas, V., Lewis, M. A., & Rudd, R. (2017). Improving low health literacy and patient engagement: A social ecological approach. *Patient Education and Counseling*, *100*(1), 8–13. doi: 10.1016/j.pec.2016.07.007 27475265

[CIT0044] Mockford, C., Staniszewska, S., Griffiths, F., & Herron-Marx, S. (2012). The impact of patient and public involvement on UK NHS health care: A systematic review. *International Journal for Quality in Health Care*, *24*(1), 28–38. doi: 10.1093/intqhc/mzr066 22109631

[CIT0045] Offit, P. A. (2008). *Autism*’*s false prophets: Bad science, risky medicine, and the search for a cure*. New York, NY: Columbia University Press.

[CIT0046] Ontario Ministry of Children and Youth Services. (2017). Ontario transforming autism services for children and their families [Press release]. Retrieved from https://news.ontario.ca/mcys/en/2017/06/ontario-transforming-autism-services-for-children-and-their-families.html

[CIT0047] Rodrigue, J., Morgan, S., & Geffken, G. (1992). Psychosocial adaptation of fathers of children with autism, down syndrome, and normal development. *Journal of Autism and Developmental Disorders*, *22*(2), 249–263. 1385591 10.1007/BF01058154

[CIT0048] Sandelowski, M. (1995). Sample size in qualitative research. *Research in Nursing & Health*, *18*(2), 179–183. 7899572 10.1002/nur.4770180211

[CIT0049] Semrau, M., Lempp, H., Keynejad, R., Evans-Lacko, S., Mugisha, J., Raja, S., … Hanlon, C. (2016). Service user and caregiver involvement in mental health system strengthening in low- and middle-income countries: Systematic review. *BMC Health Services Research*, *16*(1), 1–18. doi: 10.1186/s12913-016-1323-8 26931580 PMC4774091

[CIT0050] Strauss, A. L. (1987). *Qualitative analysis for social scientists*. Cambridge: Cambridge University Press.

[CIT0051] Strauss, A., & Corbin, J. (1998). *Basics of qualitative research: Techniques and procedures for developing grounded theory* (2nd ed.). Thousand Oaks, CA: Sage.

[CIT0052] Stroebe, M., Schut, H., & Boerner, K. (2017). Cautioning health-care professionals: Bereaved persons are misguided through the stages of grief. *OMEGA (Westport): Journal of Death and Dying*, *74*(4), 455–473. doi: 10.1177/0030222817691870 28355991 PMC5375020

[CIT0053] Valentine, K. (2010). A consideration of medicalisation: Choice, engagement and other responsibilities of parents of children with autism spectrum disorder. *Social Science & Medicine (1982)*, *71*(5), 950–957. doi: 10.1016/j.socscimed.2010.06.010 20619521

[CIT0054] Vivanti, G., & Dissanayake, C. (2016). Outcome for children receiving the early start Denver model before and after 48 months. *Journal of Autism and Developmental Disorders*, *46*(7), 2441–2449. doi: 10.1007/s10803-016-2777-6 27020055

[CIT0055] Wathen, C. N., & Harris, R. M. (2007). I try to take care of it myself.” How rural women search for health information. *Qualitative Health Research*, *17*(5), 639–651. 17478646 10.1177/1049732307301236

[CIT0056] Weiss, J. A., Whelan, M., McMorris, C., Carroll, C., & the Canadian Autism Spectrum Disorders Alliance. Autism in Canada: National needs assessment survey for families, individuals with autism spectrum disorder and professionals (1–51). Retrieved from http://www.casda.ca/wp-content/uploads/2015/12/NationalNeedsAssessmentSurvey_July-30.pdf

[CIT0057] West, R. (2005). Time for a change: Putting the transtheoretical (stages of change) model to rest. *Addiction (Abingdon, England)*, *100*(8), 1036–1039. doi: 10.1111/j.1360-0443.2005.01139.x 16042624

[CIT0058] World Health Organization. (2001). International Classification of Functioning, Disability and Health (ICF). Geneva, Switzerland. Retrieved from http://www.who.int/classifications/icf/en/

[CIT0059] World Health Organization. (2002). *Towards a common language for functioning, disability and health (ICF)* (pp. 1–23). Geneva, Switzerland: Author.

